# Nursing care for persons with developmental disabilities: Review of literature on barriers and facilitators faced by nurses to provide care

**DOI:** 10.1002/nop2.1338

**Published:** 2022-08-24

**Authors:** Nazilla Khanlou, Attia Khan, Christine Kurtz Landy, Rani Srivastava, Shirley McMillan, Susan VanDeVelde‐Coke, Luz Maria Vazquez

**Affiliations:** ^1^ Faculty of Health York University Toronto Ontario Canada; ^2^ School of Nursing Thompson Rivers University Kamloops British Columbia Canada; ^3^ Azrieli Adult Neurodevelopmental Centre Centre for Addiction and Mental Health (CAMH) Toronto Ontario Canada; ^4^ Autism Services Kerry's Place Aurora Ontario Canada

**Keywords:** developmental disabilities, health disparities, narrative review, nurses, nursing care, nursing competencies, nursing education

## Abstract

**Aims:**

To identify barriers and facilitators to nursing care of individuals with developmental disabilities (DDs).

**Background:**

Individuals with DDs experience health disparities. Nurses, although well positioned to provide optimal care to this population, face challenges.

**Design:**

Narrative review of extant published peer‐reviewed literature.

**Data Sources:**

Electronic databases, ProQuest and EBSCO, were searched for studies published in English between 2000 and 2019.

**Review Methods:**

Three reviewers reviewed abstracts and completed data extraction. Knowledge synthesis was completed by evaluating the 17 selected studies.

**Results:**

Emerging themes were: (1) barriers and challenges to nursing interventions; (2) facilitators to nursing care; and (3) recommendations for nursing education, policy and practice.

**Conclusion:**

Nursing has the potential to be a key partner in supporting the health of people with DDs.

**Impact:**

There is a need for specific education and training, so nurses are better equipped to provide care for people with DDs.

## 1 INTRODUCTION

Nurses have the potential to contribute statistically significantly to the health of individuals with developmental disabilities (DDs) in acute care, community settings and school settings. Yet, there are few nursing interventions and best practice guidelines focussing on nursing care for people with DDs. This leaves an important gap in our knowledge and practice of nursing care for people with DDs. Moreover, there are wide variations in nursing education and care in developmental disabilities internationally; for example, in the United Kingdom Learning Disabilities Nursing education is offered at the university level and the role of Learning Disability Nurse exists, but there are no similar parallels in Canada. This leads to unequal pathways of nursing care across settings.

Individuals with developmental disabilities experience a constellation of health problems complicated by absence of inclusive health promotion, accessible health care and inequitable distribution of social determinants of health (Marks & Sisirak, 2017). Research from Canada, the United States, the United Kingdom and Australia has highlighted that people with DDs are poorly supported by healthcare systems and services (Fisher, 2004; Krahn et al., 2006; Scheepers et al., 2005; Sullivan et al., 2011). Providing optimal care for this population is often perceived by healthcare professionals as difficult because of clients' disability‐related social, environmental, cultural, cognitive, behavioural and communication needs (Cheak‐Zamora & Teti, 2015; Raemy & Paignon, 2019). Nurses working in healthcare settings, community settings and school settings have reported gaps in education and training to address the complex and varied needs of people with DDs (Betz, 2007; Fisher et al., 2020; Ndengeyingoma & Ruel, 2016; Raemy & Paignon, 2019; Singer, 2013; Weiss et al., 2010). It is important that nursing care for persons with developmental disabilities be enhanced and standardized with a concentrated focus through nursing education and research.

### 1.1 Background

Developmental disabilities can encompass limitations in multiple aspects of functioning, including in intellectual capacity (intellectual and executive functioning), adaptive skills (impairments in language and communication, behavioural, emotional regulation) and physical functioning. These limitations begin in childhood and are usually life‐long affecting areas of major life activity including ability to self‐care and live independently as adults (CDC, 2017; Services and Support to Promote the Social Inclusion of Persons with Developmental Disabilities Act, 2008, S.O. 2008, c. 14, 2019). DDs include neurodevelopmental disorders, such as autism spectrum disorder (ASD), Down syndrome, fragile X syndrome, intellectual disabilities and cerebral palsy (American Psychiatric Association, 2013; Thapar et al., 2017). The 2017 Canadian Disability Survey (Morris et al., 2018) reports one in five (22% or 6.2 million people) Canadians (15 years and older) live with one or more disabilities (e.g., mental health, mobility, flexibility, mobility and pain impairment), and 315,470 (or 1.12%) Canadians lived with a DDs. In just five years the number of individuals (15 years and older) with a DDs has almost doubled; in 2012, about 160,500 persons lived with a DDs (Arim, 2015) and in 2017 approximately 315,470 persons reported living with a DDs (Statistics Canada, 2020).

Compared to the general population young people with DDs experience more comorbid conditions (Schieve et al., 2012) and have a greater onset of chronic health conditions and higher rates of morbidity in later life (Hamdani & Lunsky, 2016; Thomas et al., 2011). They also face difficulties in self‐management of health (Cheak‐Zamora & Teti, 2015), adhering to treatment (Cooper et al., 2006), voicing their care concerns (Boylan & Ing, 2005) and have reduced capacity to manage challenging situations such as loss and separation from family (Janssen et al., 2002; Tyrer et al., 2006). Health inequities further prevent young adults with DDs from accessing health resources (Emerson et al., 2012; Lin et al., 2019; Marks et al., 2008) and affordable healthcare and mental healthcare services (Crane et al., 2019).

Nurses are well positioned to provide direct and coordinated optimal care to individuals with DDs at all healthcare levels (Giarelli & Gardner, 2012; Mandal et al., 2020) but are not fully equipped to take on this active care role (Anderson et al., 2013; Cieza, 2015). Nurses have reported difficulties in providing care for individuals with DDs due to their clients' communication, social, cognitive, behavioural and physical impairments (Hahn, 2003; Smeltzer et al., 2012). More than ever before the number of individuals with DDs needing health care has increased in acute care, primary care, long‐term care and in the community (Harris‐Kojetin et al., 2016). Without targeted nursing education and training focussed on appropriate and effective health care for people with DDs, who experience a wide range of barriers and life‐stage specific healthcare needs, nurses will encounter increasing challenges in providing high‐quality care tailored to the specific individual needs of clients with DDs (Chiri & Warfield, 2012; Gardner et al., 2016; Giarelli & Gardner, 2012; Johnson et al., 2012). In this paper we present findings from a review of published peer‐reviewed literature on effective training and education approaches for nurses caring for persons with DDs.

## 2 METHODS

### 2.1 Aim

Our overall aim was to identify the barriers and facilitators to nursing care of individuals with DDs and provide recommendations. The main objectives of our review were to identify:

- Research evidence on nursing strategies and interventions for health promotion and health care of individuals with DDs;
- Knowledge gaps in nursing care that promote the health of individuals with DDs; and
- The barriers and facilitators to nursing interventions in care of people with DDs.

### 2.2 Design

We used a narrative literature review approach (Green et al., 2006) to address the issue of how to better prepare nurses to practice quality care of people with DDs. Narratives reviews, also known as unsystematic narrative reviews, are not meant to be systematic or comprehensive searches of all relevant literature on an issue (Davies, 2000). They are used to present a broad perspective and narrative syntheses of previously published information on an identified issue and recommendations to address an issue (Green et al., 2006) and to discuss a theoretical point of view (Bernardo et al., 2004). Narrative reviews have been used as educational tools in the continuing medical education field (Bernardo et al., 2004).

### 2.3 Search methods

Peer‐reviewed literature published from January 2000 up to January 2019 were reviewed to assess the knowledge on nursing strategies and health promotion interventions for individuals with DDs.

### 2.4 Literature search strategy

**FIGURE 1:**
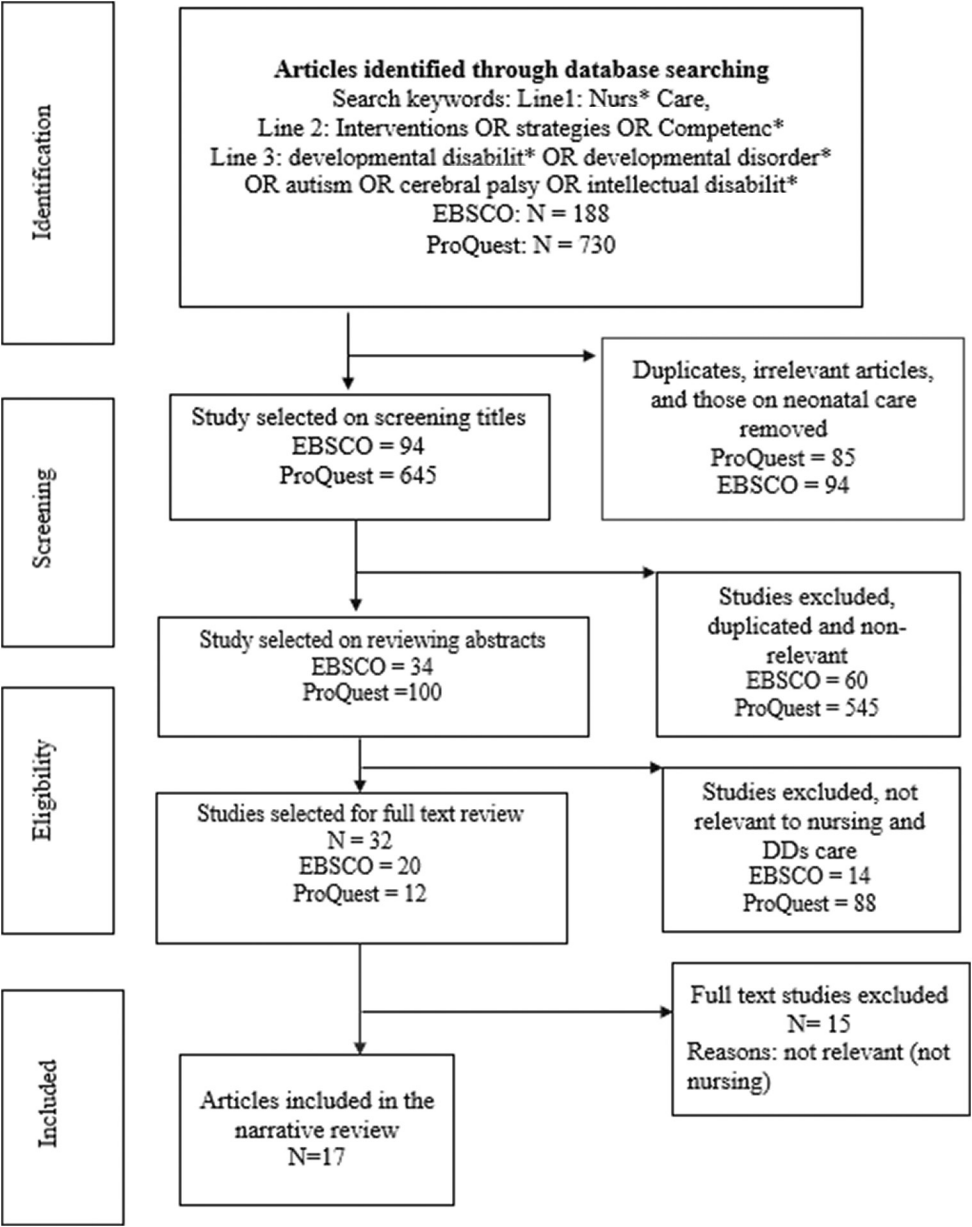
Literature review search strategy

A librarian specialized in health literature searches was consulted to assist with the development of the search strategy. The search was conducted using two electronic databases: ProQuest (included all databases) and EBSCO (included all databases), which focus largely on nursing literature. We used the following search terms: Line1: Nurs* Care; line 2: Interventions OR strategies OR Competenc*; and line 3: “developmental disabilit*” OR “developmental disorder*” OR autism OR “cerebral palsy” OR “intellectual disabilit*” across all databases. Figure 1 illustrates the search strategy undertaken. Two reviewers (2nd author and a research personnel) ran the data base searches and selected articles that met eligibility criteria. All types of studies (including reviews and case studies) that focussed on nursing strategies and health promotion interventions for children, youth, elders and adults with DDs in Canada, USA, UK, Australia and New Zealand were included given their similarities in English language and economies. Studies that did not focus on nursing care of individuals with DDs care and not situated in Canada, USA, UK, Australia and New Zealand were excluded. The initial title search yielded 94 studies from EBSCO and 645 studies from ProQuest databases. After duplicate and non‐relevant studies were excluded 34 studies were selected from EBSCO and 100 from ProQuest for abstract review. Three reviewers (2nd author and two research personnel) reviewed the abstracts for selection of articles for full text review. The involvement of the third reviewer was to help resolve disagreements. They discussed the selected articles after re‐reviewing inclusion and exclusion criteria and disagreements resolved till consensus was reached on selected studies.

### 2.5 Search outcomes

The PRISMA flow chart in Figure 1 illustrates the search strategy for the narrative review from initial identification to selection of eligible studies. A total of 32 studies (12 studies from ProQuest and 20 from EBSCO) were selected for full text review by two reviewers (2nd author and last author). Among these 17 selected articles met the eligibility criteria for the narrative review. The excluded articles did not focus on nursing strategies on the care of individuals with DDs. Those focussing on neonates with DDs were also excluded.

### 2.6 Quality appraisal

The researchers consulted the Preferred Reporting Items for Systematic Review and Meta‐Analyses (PRISMA) extension for scoping reviews statement (Tricco et al., 2018) as a guidance for a thorough examination of the literature and in the limits of a narrative review that does not require a quality appraisal of studies, appraisal of the process and audit of discarded studies. To ensure credibility of studies, the review incorporated several elements of a systematic review including: (1) enhanced reliability by employing three researchers to review the abstracts and full text studies, and then compare/ contrast selected studies, and extracted information. Further, the knowledge synthesis was reviewed by all authors; and (2) using a pre‐defined inclusion and exclusion criteria to select or discard studies. For example, studies were excluded if they did not focus on nursing care of individuals with DDs care, or examined nursing care of neonates with DDs, or were in countries other than Canada, USA, UK, Australia and New Zealand. The limits of narrative summary in assessing quality of studies have been further discussed in the limitations section.

### 2.7 Data abstraction and synthesis

Data was extracted onto an Excel file. Discrepancies between the reviewers were discussed and resolved. The specific study characteristics gathered included study goals, research design, description of participants and type of disability, tools and strategies used, and study recommendations. The results of individual studies were then analysed iteratively by two authors to determine major themes in relation to issues, tools, strategies and recommendations. Table 1 presents summary of the characteristics of each study.

**TABLE 1:** Characteristics of studies

References and year	Study aim and objectives	Research design	Location and study participants	Type of disability	Tools, strategies used	Findings and challenges	Recommendations
*Review studies*							
1. Balakas et al. (2015)	To develop an adaptive care plan (ACP) and screening tool for patients with challenging behaviours as an intervention to improve the surgical experience for the children and their families	Review of published studies on screening tools and examining those from two other hospitals	Hospitals in the United States 68 families Paediatric and surgery nurses Other staff members in the perioperative area	DD	The team developed a specific adaptive care plan (ACP) and an Adaptive Care Screening Tool A trial of the ACP was given over 6‐month The interventions included in the ACP were Environment (rooms design, lighting, music, tactile objects); Decreased wait times Staffing (providing a consistent nurse, decreasing number of healthcare providers) Education of staff Enhanced communication; proactive care planning Door symbols People first language Additional resources Parents' involvement	A brief evaluation tool was used to determine the effectiveness of the ACP Parents were asked to rate their experience, 33 (48.5%) of 68 parents who returned the survey rated their experience as a five (care exceeded expectations) and 45 parents (67%) indicated either a score of four or five One in the scale indicated expectations were not met Concerns related to a lack of consistency among caregivers, difficulty maintaining support from some staff members due to the increased effort required, inconsistent identification of patients who would benefit from an ACP, ability to always staff appropriately to support delivery of an ACP and lack of ideal physical space for patients with sensory challenges	Re‐education effort provided the clinic staff with an opportunity to learn more about the patients they are caring for Use of an adaptive care planning tool can alert the nurse to a child's specific needs Development of a multidisciplinary team is necessary
2. Betz (2007)	To review salient issues that adolescents with DDs face as they transition to adulthood	Literature review	United States Adolescents with DDs Nurses	All DDs including ID	Nurses can apply the IDEA model of assessment to nursing practice IDEA model: the nurse explores the adolescent and statistically significant other goals, interests, needs and preferences for the future Following assessment, a transition plan is developed based upon identified needs that address the adolescent's goals in the domains of health care, training, employment and community living	Comprehensive and empirically based service models are needed in health care to assist adolescents with DDs manage their transition from paediatric to adult health care settings Adolescent's verbal input beyond yes‐and‐no responses enhances student participation in the transition process	Models of health care transition programs for adolescents with DDs are in their early stages of development and implementation Empirically based transition strategies need to be developed and implemented in health care settings to assist this population of youths in making successful transitions to adulthood
3. Cockburn‐Wells (2014)	To review the literature on the causes and effects and management of constipation among people with severe learning disabilities	Literature review	United Kingdom Adults with severe learning disabilities	Learning disabilities	Review of literature Holistic approach	A more holistic approach involving improvements to clients' diets and lifestyles and to carers' understanding of the risk factors specific to this patient group, should be adopted Community learning disability nurse as a transformational leader is important Most adults with severe learning disabilities live in supported accommodation, care staff must be aware of the risks associated with constipation Staff do not have any specialist knowledge of constipation thus deficiencies in providing adequate diets, encouraging exercise and positive role modelling as carers	Carers should adopt a holistic approach to the management of constipation Community learning disability nurses should help to identify and manage the condition and provide carers with relevant information and encouragement
4. Friese and Ailey (2015)	To describe a programme developed to support nursing care for specific standards of care for people with ID	Standards program review	United States Outpatient and inpatient and emergency settings	People with ID and DD	Survey 1: hospital staff concerns‐communication, dealing with behaviour of patients, assess their pain and plan discharge Survey 2: nurses specializing in ID—perception of hospital staff's, understanding of: their clients' communication patterns, altering environment to make patients less frightened or more calm assessing clients' levels of functioning A literature review: university academic medical centres about any existing specific standards of care on benchmark of nursing standards of patient care for people with ID	The standards cover nursing interventions for the provision of safe and accessible environments, to enhance communication They also address collaboration with and support for caregivers The nurses' confidence in care provision for this clientele increased on taking the educational module Online educational programme was also developed for online support for nurse	Strategies to improve care for people with ID integrated with improved data collection and electronic medical‐record systems documentation
5. McIntosh et al. (2018)	To explore school nurses increasing the compliance of hygiene routines for students with autism spectrum disorder	Literature review	United States School nurses	Children with ASD	Review of literature	To reduce and manage unwanted behaviours (attention seeking behaviour, screaming, yelling and foul language), a school nurse can use extrinsic motivation and reinforcement techniques	Social story (gives context in a picture voice to an experience) can be used in the clinic to help children with ASD understand what will happen for particular visits to make it physically, socially and emotionally safe It can be easily adapted to prompt the child with ASD to complete essential tasks
6. Sheerin (2008)	To identify the diagnoses and interventions that are employed by intellectual disability nurses	Review of professional literature	Ireland	Developmental disabilities, learning disabilities and intellectual disabilities	Review of literature	Examination of the sources yielded 30 foci for nursing intervention and 58 interventions The study highlighted the developments leading to the standardization of terminology employed (the nursing diagnoses communicate the professional judgements that nurses make) to facilitate communication between nurses and other professionals The core interventions of DDs nursing were categorized as psychological basic end complex, behavioural, safety, family, health system and community	Further inquiry to determine application of nursing diagnoses and interventions in different care settings
*Quantitative studies and case studies*							
7. Drake et al. (2012)	To evaluate the nurse's perception of the effectiveness of a coping kit intervention for children with DDs at increased risk for challenging behaviours	Quantitative study post‐test design	United States 24 nurses from 3 inpatient units and the emergency room with experience caring for a children and youth with DDs age 2 to 18 years	Children and youth with DDs including ASD, Down syndrome, neurological condition and other DDs	A coping kit with simple communication cards, social script book and distraction items (toys) was used in children with ASD and other DDs undergoing procedures in the hospital The kit enhanced communication and distracted them	The nurse used the kit for distraction during a procedure (50%), communication to prepare for a procedure (4.2%) and for other uses (50%), to reduce anxiety due to diagnosis or multiple hospitalizations or distraction during assessments and while performing vital signs Noticeable change in the child's behaviour was observed after items were given from the coping kit A calmer child may help decrease the anxiety of the nurse caring for the child as well	Effective ways to support children with behavioural challenges while in the hospital need to be sought Uptake of coping kit as a part of a package of interventions can help alleviate anxiety for the hospitalized child with behavioural challenges
8. Kirby and Hegarty (2010)	To examine proficiency, motivation and knowledge about breast cancer screening and awareness of nurses working in an Intellectual Disability setting. Additionally, the study aimed to examine and establish associations between nurses' personal and professional breast awareness practices	Quantitative descriptive design study	Cork Teaching Hospitals, Ireland Intellectual Disability 200 female nurses	Persons with ID, age 20–60 years	Data were collected using the “Modified Toronto Breast Self‐Examination Inventory” (MTBSEI) The questionnaire contains 3 scales, which assess: Proficiency Motivation Knowledge, about breast cancer and breast cancer screening modalities and additionally assesses frequency of breast self‐examination practices	Majority of nurses do not promote breast awareness and screening for women with intellectual disabilities There were deficits in nurses' personal knowledge, skills and practices in breast awareness and screening Nurses demonstrated uncertainty about their level of proficiency in the detection of personal breast anomalies 92.5% nurses required additional support in breast care only 8.5% (9) nurses received additional instruction or support in the previous 12 months Key challenges in relation to the promotion of breast awareness were absence of policies and guidelines, the women's limited cognitive functioning, communication difficulties, difficulties in attaining consent and legal and ethical issues	There is a need to support nurses with ongoing education in relation to breast awareness, in order that breast awareness be promoted in clinical practice A multifaceted approach is required in order to develop and maintain health promotion interventions with specific regard to breast awareness and screening Nurses working in intellectual disability settings require specific education programs
9. Melville et al. (2005)	To measure the attitudes, knowledge, training needs and self‐efficacy of practice nurses in their work with people with Intellectual disability	Cross sectional study	Greater Glasgow, Scotland 201 primary health care practice nurses	ID	Survey on knowledge, previous training on the topic of intellectual disabilities and self‐efficacy	86% had experienced specific difficulties during appointments Practice nurses scored highly on self‐efficacy Lack of training specific to DDs primary‐care nursing and the knowledge gap indicates that targeted training initiatives are required	Targeted training of nurses would play an important role in enhancing primary health of individuals with DDs A broad range of models and methods can be used for training initiatives such as written training materials, skills workshops and computer‐based distance learning packages
10. Scarpinato et al. (2010)	To explore the challenges that patients with ASD face when hospitalized and to provide assessment strategies and plan‐of‐care suggestions for nursing caregivers	Clinical case studies and scientific literature evidence	Hospital, United States	ASD	Clinical case studies	Not specified	Providing nurses specific, focused resources supports consistent care and helps ensure a safe environment for both patients and nurses Whether part of the inpatient or outpatient setting, planning and coordinating care with a multidisciplinary team is essential in providing resources, treatment and follow‐up
*Qualitative studies*							
11. Halpin and Nugent (2007)	To identify generic health visitors' perceptions of their role with families where children may have ASD	Small‐scale qualitative inductive study	Primary care NHS trusts, United Kingdom 11 health visitors	ASD	Interviews	Participants felt: they should have expertise in child development they had a role to play in identification of children who might have ASD they would appreciate and attend training on ASD if offered their competence to know when a child might need assessment for an ASD was inadequate their role with families where children may have ASD to be one of identification and ongoing support	Individual work with families is important and appropriate Training is needed Planning for the development of a keyworker service for children with complex needs should include health visitors
12. Hemsley et al. (2012)	To investigate nurses' expressed concepts of “time” in stories about communicating with patients with DDs and complex communication needs in hospital	Qualitative study	Hospital in Australia 15 hospital nurses from a range of wards	DD and complex communication needs	In‐depth interviews Interview topic guide was developed in consultation with an expert reference group: Consisting of 2 speech pathologists working with adults with ID and adults with cerebral palsy One adult with cerebral palsy who had been hospitalized in the past 2 years A family carer Disability support worker Disability liaison nurse Hospital nurse	Time was perceived by nurses as both an enemy and friend for improving communication Nurses cited “lack of time” as a barrier to effective communication Time as a facilitator to successful communication, investing extra time and to applying a range of adaptive communication strategies to establish successful communication Time as a barrier to avoiding direct communication and preferring that family or paid carers communicated on behalf of the patient Nurses who perceive that communication takes too long may avoid communication and miss opportunities to improve communication through increased familiarity with the person's communication methods Those who take time to communicate narrate applying a range of strategies to achieve success in basic needs communication Nurses' attitudes towards patients with DDs and complex communication needs may influence their perceptions of time in their encounters and methods of communicating with these patients	Extra time to communicate with patients is useful if nurses are confident and competent in using a range of adaptive communication strategies Nurses who cite time constraints as reasons for not communicating directly with patients need to be aware that patients view avoidance of communication as a reflection of negative or discriminatory attitudes towards them Nurses need to remind patients and their families to bring existing communication aids to hospital and hospital policies must support the safe storage and use of these systems in the hospital setting
13. Macdonald et al. (2018)	To explore perceptions and experience of nurses delivering an anticipatory health check for adults with ID	Nested qualitative study in an RCT	11 General Practices located in NHS Greater Glasgow and Clyde, Scotland, United Kingdom 11 practice nurses	ID	Intervention was health checks and standard care undertaken by the practice nurse (IDs nurses provided support when necessary) controls received only standard care	The intervention was superior to standard health care, the adjustments nurses made may not have maximized potential benefits to patients Increasing training could further improve the benefits that health checks provide for people with IDs	Further studies to examine gains of mandatory training Evaluate the two methods of health check delivery through clinical trials and cost‐effective trial
14. Narayanasamy et al. (2002)	To analyse how nurses caring for people with learning disabilities respond to clients' spiritual needs	Qualitative approach incorporated the critical incident technique	Residential community, United Kingdom 10 learning disability nurses	Learning disability	Participants encouraged to reflect and provide written critical incidents of their experience of spiritual care in relation to four topics: When and how they recognized that clients had spiritual needs How they could identify spiritual needs What they did to help their clients The effects of their actions on the clients	Cues from clients about their religious beliefs and practices led nurses to formulate care plans, which characterized sensitivity and consideration to their clients' religious needs Nurses who identified clients' spiritual concerns in non‐religious terms used a more personal approach (client centredness) Engagement at a personal level with clients helped them to identify emotional tensions and turmoil Nurses giving counselling support to clients helped overcome their spiritual distress Nurses more attuned to recognizing religious needs than they are to recognizing more general spiritual needs (such as the search for meaning and purpose, and a need for love and security), which could have been overlooked Nurses identified that the outcomes of their spiritual care interventions had therapeutic effects on clients, families and also on themselves on most occasions	Faith and trust in nurses produce a positive effect on people with learning disabilities and their families Spiritual care interventions promoted mostly a sense of wellbeing
15. Ndengeyingoma and Ruel (2016)	To explore nurses' representations of caring for people with ID, intervention strategies they currently use, and to identify needs to ensure quality care	Qualitative descriptive study	Canada, Gatineau centre for health and social services 18 nurses from hospital and community settings	ID	Semi‐structured interviews using thematic analysis	Informational needs in relation to best practice intervention strategies and as coherent directives to promote continuity of care Nurses concerned about their ability to recognize nature and complex needs of patients with ID, promoting quality care through management of behavioural and communication challenges, coping with the perceived lack of time and organization in the work environment Nurses identified their learning needs‐ relational and informational in nature	Nurses should be educated to adapt Adoption of specific guidelines, protocols and care programmes can facilitate, promote the continuity of care Many contextual and practical elements that require improvement to ensure the security of these patients, some expressed more interest than others
16. Singer (2013)	To explore the perceptions and challenges of school nurses working with students with intellectual and DDs	Qualitative study	Public schools (elementary and high school) in Northeast part of the United States 8 baccalaureate nurses	Students with intellectual and DDs	Personal interviews, observations and field notes	School nurses lacked education in disability studies and encountered difficulties with communication, screenings in students with DDs and completing health assessments More education and standardized tools would help nurses school‐based practice and to enhance their students' health care Assistive technology such as augmentative assistive communication (AAC) was often not used maybe due to a lack of training, insufficient expertise or lack of access to equipment	All nurses must be equipped with education and training to have the ability and tools available to accurately assess pain in students who have DDs, be able to apply alternative communication methods including assistive technology
17. Zwaigenbaum et al. (2016)	To explore the perspectives of health professionals (doctors and nurses) caring for children with ASD in the emergency department and to determine the strategies to optimize care	Qualitative study, grounded theory design	Canada Emergency department high acuity paediatric health sciences centres 10 physicians and 12 nurses were interviewed individually	Children with ASD under 18 years	Interview guide collected data on doctors and nurses experiences providing care to the child with ASD in ED encounters, factors challenged and facilitated effective care, strategies or recommendations based on current or past experiences to improve ED care for children with ASD and their families	Challenges: (1) increased challenges working with older children‐youths, were physical larger and with increased aggressiveness (2) sensory triggers caused distress for patients and made care provision more difficult. (3) competing demands in the ED prevented ideal care Facilitators: Using communication strategies, involving parents, providing a calming environment and preparing doctors and nurses through training and teamwork, collaboration	Education and training on educational approaches in working with this population, communication and collaboration with parents Partnering with expert Process change Patients with DDs receive faster service and be triaged quicker Need for changes to hospital space and available resources

*Note*: Study participant is defined as an individual who participates in the study or a person on or in respect of whom any study activities are performed. Tools refers to how data was collected and strategies to the interventions. In the United Kingdom “learning disabilitys” refers to ID.

Abbreviations: ASD, autism spectrum disorder; DD, developmental disability; DDs, developmental disabilities; ED, emergency department; ID, intellectual disability; RCT, randomized controlled trial.

## 3 RESULTS

Among the 17 eligible studies, six were qualitative, one was mixed methods (nested qualitative study in a randomized controlled study), three were quantitative (two descriptive and one interventional study), one was a clinical case study, and five studies were literature reviews, and one was a program review. The themes identified from our literature review have been organized by overarching themes as: (1) Barriers and challenges to nursing interventions in care of people with DDs; (2) facilitators to nursing care in promoting the health of individuals with DDs; (3) and emerging recommendations for nursing education, policy and practice.

### 3.1 Barriers and challenges to nursing interventions in care of people with DDs

Our narrative review found nurses experienced overlapping health systems‐based challenges to providing optimal care to people with DDs, which included time constraints and insufficient staffing, communication challenges and insufficient education and training.

#### 3.1.1 Time constraints and insufficient staffing

Nurses reported time constraints in providing accommodations and having additional responsibilities when caring for people with DDs that may include undertaking health checks, providing compassionate care and relaying health information to clients and their caregivers on medical procedures and examination (Ford, 2020; Hemsley et al., 2012). Due to time constraints nurses often avoided direct communication with the patient and depended more upon family carers for communication about procedures and treatment (Hemsley et al., 2012). Not interacting with patients with DDs hindered the development of effective relationships between nurses and their clientele. Shortage of time was also noted to be a barrier to conducting periodic health checks of individuals who have DDs. These comprehensive health assessments, conducted as preventive and diagnostic measures, in primary‐care clinics have been shown to be beneficial for health of people with DDs. The health checks for one patient with DDs typically took 48 min to carry out (Macdonald et al., 2018). The time required to administer the health check was centred around the operational impact on how this intervention impinged on nurses' everyday workload and practice particularly if dealing with larger numbers of patients (Macdonald et al., 2018). Ndengeyingoma and Ruel (2016) identified that nurses had insufficient time to intervene adequately in situations concerning people with DDs in the emergency room and hospital ward, particularly when care was urgent. Patients with DDs required more time for communication for gathering medical histories for their needs to be specified and procedures to be explained (Ndengeyingoma & Ruel, 2016). Patients with DDs often need more time explaining but, as time is limited, they may feel rushed and not fully absorb the information, which may cause elevated emotions that they have challenges dealing with (Ndengeyingoma & Ruel, 2016). Thus, rushing urgent care prevented organized care and led to inadequate care approaches, such as use of restraints and sedative medication to prevent injury to clients (Ndengeyingoma & Ruel, 2016).

Insufficient staffing magnified the problems associated with managing people with DDs. Nurses reported that because of insufficient staffing providing people with DDs the necessary accommodations were challenging (Macdonald et al., 2018). The nurse—patient ratio did not allow nurses to allocate more time to patients with DDs, thereby respecting their varied needs (Ndengeyingoma & Ruel, 2016). Nurses were expected to closely monitor and manage patients with DDs concurrently with their regular duties without any reduction in workload. Moreover, during night shifts fewer healthcare providers knowledgeable in ASD were available (Ndengeyingoma & Ruel, 2016). In the emergency department nurses were under constant stress to complete tasks and procedures and their anxiety was heightened when caring for a child with DDs (Drake et al., 2012).

#### 3.1.2 Communication challenges

Nurse reported communication challenges with individuals with DDs, their caregivers and care staff including other nurses, healthcare providers and multidisciplinary teams (Melville et al., 2005). For example, in healthcare settings, because of difficulties managing communication challenges with people with DDs, nurses had difficulty responding to their patient's needs and explaining the intervention or procedure (Ndengeyingoma & Ruel, 2016). Families believed that nurses deliberately avoided communication. They perceived this as a negative and discriminatory attitude towards their family members with DDs, which impacted the quality of hospital care their family members with DDs received.

Enhanced communication when caring or supporting people with DDs is crucial at all levels of health care (Hemsley et al., 2012). In the emergency department nurses had to pay close attention to the non‐verbal cues of children with DDs, specifically those with limited verbal ability (Zwaigenbaum et al., 2016). In the absence of a family caregiver/ parent, the experts on their child's needs, preferences and aversions, the use of Augmentative and Alternative Communication (AAC) (including gesture or signing systems, interpreters, communication boards or speech generating devices) by persons with DDs to share their needs reduced nurses guessing their needs and was also more comfortable for the patients (Hemsley et al., 2012). Nurses believed that investing time in learning skills in communication would lead to better assessment, and ultimately save time (Ndengeyingoma & Ruel, 2016). In hospital settings at shift change communication between nurses were typically not sufficient to convey and comprehend information about patients with DDs' medical condition, behaviour, routines and functional abilities in activities of daily living, such as eating preferences (Friese & Ailey, 2015). Communication was hampered by time constraints, and by level of comfort between the nurses and person with DDs (Singer, 2013). In school settings nurses experienced communication challenges with students with DDs, who were not able to articulate their needs. Nurses had difficulty explaining procedures to them and making accurate assessments. Moreover, they were unable to assess if the student had understood their instructions, because not being provided the baseline information on the Individualized Education Program (IEP) they could not assess the developmental levels of their students (Singer, 2013).

#### 3.1.3 Insufficient education and training on supporting individuals with DDs

Nurses tended to be more comfortable if they had previous experience caring for patients with a disability and/ or became familiar with persons with DDs over time (Singer, 2013). Several studies have shown that education and training on disability provided in nursing schools and during continuing education and professional career development were insufficient. A United Kingdom based study on the training needs of primary health care nurses (*N* = 201) found 86% of practice nurses had communication difficulties during appointments with people with DDs and only 8% of had received training in communicating with this clientele (Melville et al., 2005). In another study nurses in Ireland promoting breasts awareness programs were found to have limited knowledge of women with DDs' cognitive functioning and communication abilities (Kirby & Hegarty, 2010).

Professional development programs to educate nurses on specialized health care for children and adults with DDs' diverse needs were found to be insufficient. Registered Nurses in the United Kingdom with additional training in community public health, also known as Health Visitors, had difficulty identifying the health needs of children with ASD and providing them ongoing support because they lacked education and training on the care of people with ASD (Halpin & Nugent, 2007). Similarly, care staff for adults with severe DDs living in supported accommodation did not have any specialist knowledge in the care of people with DDs, including the risks associated with constipation, a common ailment in this clientele (Cockburn‐Wells, 2014). On a similar note, nurses with several years of nursing experience who transitioned to school nurses had not received any orientation on students with DDs nor were they included in training or staff meetings about this clientele (Singer, 2013). Thus, the school nurses encountered difficulties with communication, screenings and completing health assessments of these students (Singer, 2013).

A study from Quebec, Canada, noted that hospital nurses' learning needs were informational in nature and relational to best practice intervention strategies, ensuring consistent high‐quality personalized care to individuals with DDs (Ndengeyingoma & Ruel, 2016). The strategies included nurses' ability to recognize features of DDs, identify their clients' needs, and organize the continuity of care. The 18 nurses who participated in the study reported that they did not feel confident about their ability to recognize the characteristics of DDs if a patient presented with the condition. They also were unable to recognize the complex needs of patients with DDs and as a result their clinical evaluations of their patients were often incomplete (Ndengeyingoma & Ruel, 2016). Health visitors working with families of children with ASD felt they: did not have adequate expertise in child development; lacked competence in their role in identifying children who might have ASD; and would attend training on ASD if offered (Halpin & Nugent, 2007). Registered Nurses at a children's hospital participating in a study examining the effectiveness of a coping kit to manage challenging behaviour in children with DDs, including ASD, reported programs that can train nurses in behaviour management skills were often unavailable in their work settings (Drake et al., 2012). Inadequate training and limited resources (such as time constraints) can heighten nurses' challenges in providing care and support of children with DDs (Zwaigenbaum et al., 2016). Findings from a study examining the use of an evaluation tool (the Adaptive Care Screening Tool) to prepare children with DDs for surgery showed that developing a multidisciplinary team and re‐educating the clinic staff were essential for effective perioperative care for this clientele (Balakas et al., 2015).

Kirby and Hegarty (2010) found all grades of nurses (*n* = 200) in Southern Ireland experienced challenges in promoting breast awareness and screening for women with DDs. Nurses reported inadequacy in their skills on breast self‐examination, uncertainty in their proficiency in the detection of breast anomalies, and difficulties addressing legal and ethical issues, and obtaining consent from women with DDs.

### 3.2 Facilitators to nursing care in promoting the health of individuals with DDs

Our review identified several factors, which facilitated nurses in providing enhanced care to people with DDs. These include the use of tools and focussed resources, nursing strategies to manage challenging behaviours and using strategies that improve collaboration with family caregivers and healthcare teams.

#### 3.2.1 Tools and focussed resources for nursing care of individuals with DDs

The need for a comprehensive “health service model” to support the psychosocial, medical and educational/ vocational needs of adolescents with DDs in their transition from child‐focussed to adult‐focussed healthcare system in the United States of America (USA) has been identified (Betz, 2007). Nurses must have knowledge of their client's transitional needs, so they can develop a youth‐centred transition plan along with members of the school's IEP team that addresses their comprehensive health needs (Betz, 2007). The USA based IDEA (Individuals with Disabilities Education Act^1^) model of assessment to nursing practice allows nurses, with facilitated verbal input of youth with DDs and their caregivers, to identify interests, needs and preferences of transitioning youth for the future, and to develop a transition plan aligned to the youth's goals in the areas of health care, training, employment and community living (Betz, 2007). On the same note, school nurses suggested that access to standardized assessment tools and more education on assessment would help their practice and to providing better health care to students with DDs (Singer, 2013).

Focussed resources can support and enhance consistency of nursing care and contribute to a safer environment for both nurses and their patients with DDs (Scarpinato et al., 2010). A study examining the benefits of nurse conducted routine health checks in the primary care of people with DDs (the intervention), versus standard health care, found the intervention was most successful with patients whose problems or issues were recognized by nurses, and that further training of nurses would maximize potential benefits for the patients (Macdonald et al., 2018). A written critical incident technique in the United Kingdom helped nurses to pick up cues from their clients with DDs about their religious beliefs and practices and to formulate care plans using a client‐centred approach. This approach helped to identify emotional tensions and turmoil and provide clients the appropriate counselling support to address their spiritual needs (Narayanasamy et al., 2002).

#### 3.2.2 Nursing strategies to manage challenging behaviours

It can be particularly difficult for nurses to provide essential care and treatment for people with DDs with challenging behaviours (Zwaigenbaum et al., 2016). The unfamiliar setting of the emergency department, the “hustle and bustle”, noise and being seen by multiple unfamiliar health care providers can be disturbing for a child with ASD and cause statistically significant stress and anxiety for people with DDs. Further, negative experiences at the hospital or health care visit can potentially impact the behaviour of child's future visits (Zwaigenbaum et al., 2016). In addition, the increased sensitivity to touch of some people with DDs makes it more difficult for nurses to complete clinical examination and diagnostic procedures.

Nurses in healthcare and school settings can adopt techniques to calm children and youth with DDs' behaviour and improve treatment delivery (Drake et al., 2012; McIntosh et al., 2018). Optimizing the environment (Singer, 2013) by incorporating “warming up” techniques, moving slowly through procedures and using distraction techniques (e.g., TV, toys and video games) can potentially provide a more positive experience for a child with ASD (Zwaigenbaum et al., 2016) and also make patients with DDs feel calm and less frightened (Friese & Ailey, 2015). Changing the environment (Zwaigenbaum et al., 2016), using proactive strategies (such as creativity, sensitivity and awareness) and coping kits can potentially alleviate anxiety and increase cooperation in the hospitalized child with ASD (Drake et al., 2012). These strategies help to reduce, prevent and manage challenging behaviours at initiation of every health care visit (Friese & Ailey, 2015). While working with families of children with DDs, availing the parents' expertise reaped increased care benefits (Drake et al., 2012).

The Adaptive Care Plan (ACP) was developed in USA hospitals to improve the surgical experience of children and adolescents with DDs and their families and to manage patients' challenging behaviours (Balakas et al., 2015); it provided nurses an opportunity to learn more about children and adolescents with DDs in their care and to alert them to the child's specific needs. The ACP focussed on several aspects of care including improving the care environment and staffing, educating staff, enhancing communication with clients and increasing parental involvement (Balakas et al., 2015). The ACP also provided guidance on developing and training multidisciplinary team for care of children with DDs (Balakas et al., 2015).

The use of “extrinsic motivation and reinforcement techniques” helped school nurses to promote compliance of hygiene routines for students with ASD (McIntosh et al., 2018). These techniques reduced and managed unwanted attention seeking behaviours in children with ASD. The “give‐get exchange method” used positive reinforcement (such as using verbal praise or gifting candies, stickers or allowing company of favourite toy or person) to motivate the child to do something (McIntosh et al., 2018). Other methods included giving clear, honest explanations paired with a visual aid of what is expected of the child and offering a simple choice. For example, the use of *social stories* ^2^ to make children with DDs more calm and less frightened for procedures improved patients' cooperation when care was being provided and reduced behavioural disruptions in hospitals (Friese & Ailey, 2015; Kokina & Kern, 2010). Similarly, in school settings social stories reduced unwanted behaviour and prompted children with ASD to complete essential tasks (McIntosh et al., 2018).

#### 3.2.3 Collaborating with nursing staff, healthcare teams and family caregivers

Family caregivers can be a key resource for the patient with DDs and the healthcare team including nurses (Balakas et al., 2015). Parents' involvement and the support of multidisciplinary team was key in the development and implementation of the ACP screening tool (Balakas et al., 2015). Parents have unique knowledge about their child's likes and dislikes, and what strategies to adopt to help calm their child and, therefore, are essential for care delivery in the emergency department (Zwaigenbaum et al., 2016). Showing positive regard to family caregivers for their role and knowledge about the person with DDs' health, medical history, needs and behaviour and communicating with caregivers about type of accommodations that might be beneficial for their child with DDs proved fruitful (Friese & Ailey, 2015; Halpin & Nugent, 2007). Families and caregivers require ongoing support as they are integral to the wellbeing of people with intellectual disabilities (Halpin & Nugent, 2007). While caregiving is a huge physical and psychological burden and can adversely affect caregiver's ability to provide effective care, involving parents in providing a calming environment and training teamwork and collaboration can facilitate nursing care (Zwaigenbaum et al., 2016).

By coordinating care with formal care systems and residential placement facilities nurses can support the care of youth with DDs transitioning between inpatient treatment and healthy living in the community (Ndengeyingoma & Ruel, 2016). The community DDs nurses (as transformational leaders) can play a vital role to reduce care deficiencies by coordinating holistic care and encouraging caregivers not having specialist knowledge of certain conditions (Cockburn‐Wells, 2014). A holistic approach involving improvements to adults with DDs' (living in supported accommodation) diets and lifestyles, such as exercise, and improving caregivers' understanding of the risk factors (for example, constipation), is recommended (Cockburn‐Wells, 2014). Shortfalls in collaboration among educational staff and nursing staff at schools pertaining to students on IEPs resulted in heavy reliance of school nurses on classroom staff for assistance on information on students with IEPs (Singer, 2013).

### 3.3 Emerging recommendations for nursing education, policy and practice

Table 2 summarizes six recommendations emerging from our review for education, policy and practice. These recommendations align with the guiding principles of the United Nations Convention on the Rights of Persons with Disabilities (2016) and the Accessible Canada Act Bill C‐81 (2019), which promote the full and equal participation of persons with disabilities in society. The recommendations emphasize that all nurses must be equipped with education and training to have the ability and tools to identify and conduct assessments of clients who have DDs, to apply alternative communication methods, including assistive technology and apply approaches to communicate and collaborate with parents. The findings further highlight the need for employing a nursing quality and performance improvement plan that focuses on increasing the understanding of the unique needs of individuals with DDs, improving communication between clients with DDs, their families and staff.

**TABLE 2:** Recommendations for education, policy and practice on nursing strategies and health promotion interventions

	Recommendations from studies	Recommendations from review	Cited in
Access to better and more information for nurses	Accessible (time and format) empirical and applied knowledge in caring for individuals with DDs Academics, policy‐makers and nursing educators to develop tools, frameworks and care plans, which can be used by nurses and nursing students working on the frontline with the patients Community nurses trained in care of persons with DDs can provide family carers with relevant information	Nurses are not finding time to engage in education, thus access to information needs to be “just in time” approach that can be accessed as needed Informational needs should align with best practices and promote continuity of care	Cockburn‐Wells (2014) Ndengeyingoma and Ruel (2016)
Education and training for nurses, staff and multidisciplinary team	Nurses require specialized education and training programs in DDs Re‐education efforts to provide opportunities to clinic nurses to learn more about the patients under their care Online educational module offering flexible education and training approaches (staff nurses generally have lack of time to undertake educational modules on their units)	Ensuring that this content exists in the nursing curricula Focused continuing education opportunities for nurses Online education also needs to be supported by providing time and facilitation to nurses in practice	Balakas et al. (2015) Friese and Ailey (2015) Halpin and Nugent (2007) Kirby and Hegarty (2010) Ndengeyingoma and Ruel (2016) Scarpinato et al. (2010) Singer (2013) Zwaigenbaum et al. (2016)
More collaboration between families, health professionals and staff	Collaborating with parents and involving multidisciplinary team members to reinforce the continuity of care Planning and coordinating care with a multidisciplinary team helps in providing resources, treatment and follow‐up Including the perspectives of persons with DDs and their family caregivers Care work of families of those with DDs should be integrated. This helps to establish faith and trust in nurses and produces a positive effect on people with DDs and their families providing care Education and resources for family caregivers are essential Optimize care in specialty clinics by engaging paediatric nurses who can help evaluate or provide treatment to children and adolescents (for sleep disorders, behavioural problems), help carry out educational interventions and make recommendations for referral to paediatric specialized care (for further assessment and treatment)	Reinforce the importance of engaging families in care Including families in the initial assessment process and regular review of care plans Nurses should be working with professionals such as behaviour therapists when supporting autistic persons Behaviour therapists can train, educate and work with parents in managing behaviour and techniques in the home, via ZOOM and virtual media	Zwaigenbaum et al. (2016) Friese and Ailey (2015) Balakas et al. (2015) Scarpinato et al. (2010) Narayanasamy, Gates and Swinton (2002)
Improving informal communication and interaction with patients	Simplifying language, aiming for a gentle, comforting and strength‐based relational approach, and understanding of patient's communication patterns and needs Training, peer mentoring and supporting nurses to be more confident and competent in using a range of adaptive communication strategies Dedicating more time to communicate with the child with DDs or ASD (e.g., paying attention to non‐verbal cues and assessing anxiety and stress levels and improving communication with children with ASD)	Time required for nursing care strategies needs to be recognized and planned for by nurses and the organizations delivering or providing the services	Friese and Ailey (2015) Hemsley et al. (2012)
Promoting use of standardized assessment tools	Standardized tools should be promoted to collect critical information about the children from parents Promoting use of Adaptive Care Screening Tool to enable usage and adjusting interventions to better the quality of care for those with DDs. (The adjustments include the room design, playing music, tactile objects, having a consistent nurse and promoting parent's involvement)	Standardized tools can help with accuracy of assessments and enhance health	Balakas et al. (2015) Singer (2013)
Creating a safe environment	Faster service and quicker triage for patients with DDs Changes in hospital space and available resources (altering environment can make adults and children with DDs less frightened, less anxious, safe and calmer)	Providing accessible environments	Zwaigenbaum et al. (2016) Singer (2013)

Nurses play a vital role in inter‐professional healthcare teams, supporting and advocating for clients and their families. The revised Canadian Consensus Guidelines for primary care of adults with intellectual and DDs provide recommendations and appropriate modifications to standard practice to enable family physicians and primary‐care providers improve primary‐care and health outcome of this clientele (Sullivan et al., 2018). Furthermore, tools to support primary‐care providers implement these guidelines have been developed (Surrey Place, 2020). These tools promote preventive care easily overlooked in individuals with DDs, such as immunization, screening and medication reviews. However, in Canada (where we the authors of this review are situated) evidence‐based nursing training and education on providing nursing care for this population are much needed and could have a statistically significant impact on healthcare system change in support of effective and high‐quality nursing care for people with DDs.

## 4 DISCUSSION

As brought to light by our review and reported by other researchers in the DDs field, multiple challenges impede effective nursing care of people with DDs, including communication barriers (Chew et al., 2009; Ee et al., 2021), limited time to complete tasks that require more time, dearth of staffing with the needed skill mix (Beeber et al., 2014), inadequate education and training on managing individuals with DDs (Ee et al., 2021) and need for more involvement of parents and nurses working with other professionals (Law et al., 2003) including behaviour therapists, occupational therapists (Hines et al., 2019) and speech language pathologists (Auert et al., 2012) when supporting persons with autism. Thompson and colleagues (2008) report similar findings that nurses' busyness impacted their ability to provide complete nursing care and to find or use resources. Moreover, busyness caused emotional and physical strain and sacrifice of nurses' personal time (Thompson et al., 2008).

The highest importance in the training of healthcare professionals working with people with developmental disabilities is situated in the professionals' competence experiences and comfort (Smith et al., 2021). Curricular changes (at educational institutions) are required so nurses are better equipped to take on this very important role. These include providing nurses educational and training resources and using a multi‐level and multidisciplinary approach to capacity‐building. Although developments, such as the model of specialist nursing care in United Kingdom have been observed, transformation on the education, training and recruitment of nurses in the care of people with developmental disabilities is much needed in other countries (Wilson et al., 2022).

The complexity of providing nursing care to individuals with DDs can be further addressed through organizational and workforce development. The World Health Organization emphasizes that to address health disparities it is essential to enhance the development of human resources, such as nursing workforce, which can improve access to services and supports for marginalized and vulnerable populations (e.g., individuals with DDs) (World Health Orgnization, 2016). This review has made visible the challenges and barriers in providing nursing care to people with DDs, the need for support in nursing training and education in the DDs field, and collaborative and organizational approaches to enhance nursing care. Our review further underscores that nursing interventions/ strategies for people with DDs are underdeveloped, with few practice guidelines available and developed. There is also a scarceness of research examining the effectiveness of nursing interventions to improve health outcomes and health care access for people with DDs.

A multidisciplinary and team‐oriented approach is important. For example, health professionals such as behaviour therapists are key in supporting autistic persons. They are educationally prepared to assist nurses in understanding the appropriate techniques to mitigate challenging behaviours observed in autistic persons. Parents are also key in supporting autistic persons and learning the appropriate mitigating behaviour techniques. Behaviour therapists train and educate parents in those techniques and work with them either in the home, or now via Zoom and virtual media.

### 4.1 Differential impact of the pandemic on persons with DDs

Our narrative review focussed on published literature prior to the pandemic, given the statistically significantly different pre‐pandemic health context. However, it is prudent to note the impacts of the ongoing pandemic on persons with DDs. The current COVID‐19 pandemic has magnified the gaps and disparities in health outcomes of vulnerable populations around the world. Growing evidence indicates that marginalized populations are at an increased risk of COVID‐19 related morbidity and mortality outcomes. People with mental health and developmental disabilities face multiple social and economic barriers, which further exacerbate risks to their health. There is evidence that rates of complications and death due to COVID‐19 infection have been higher among people with DDs than in the rest of the population (Shapiro, 2020) especially for those living in residential settings (Landes et al., 2021).

Pandemic‐related directives such as self‐isolation and physical distancing and intermittent provision of health care and services (Armitage & Nellums, 2020) and medical rationing (Andrews et al., 2020) have impacted this population more than the general population. Developmental disabilities nurses dedicated more time assisting people with varying DDs (and their families) in understanding and coping with the pandemic‐related social changes such as social distancing, not having visitors, changes in daily routine, not being able to attend day programs (Desroches et al., 2021). Most importantly, the pandemic has exposed that level of skilled nursing care for people receiving DD services is associated with COVID‐19 outcomes (Landes et al., 2021).

There have been calls to respect the basic human rights of people with DDs and to adopt a *disability lens* approach to COVID‐19 initiatives to population health (Spagnuolo & Orsini, 2020). The WHO (2020), the Government of Canada through the Public Health Agency of Canada (Government of Canada, 2020), Ontario Human Rights Commission (Ontario Human Rights Commission, n.d.), disability organizations and scholars have urged healthcare and public health systems to recognize, prepare and address the differentiated impact the pandemic is having on vulnerable sectors of the population (Spagnuolo & Orsini, 2020). Taken together, it is being stressed that public and health workers must ensure vulnerable groups have: (i) access to non‐discriminatory health care; (ii) continuity of caregiving support; (iii) timely access to information in accessible formats and language related to COVID‐19; and (iv) access to vital public health; and free of stigma and discrimination (such as racism, ageism, ableism) in relation to COVID‐19.

### 4.2 Limitations

We recognize several limitations to our review. First, we applied a narrative review approach and, therefore, limited our examination to readily available literature. However, we have documented the process of study selection in detail and conducted the review with several team members to enhance reliability of decision making in study selection, and information extraction and synthesis. Second, the findings are based on studies available in English only, so are linguistically specific. Third, the studies included in the review had different research designs, and we did not apply an evaluation of strength of design in study selection. However, we believe learning from across study designs is informative to enhance nursing education in the DDs field. Finally, the findings from our review are specific to several English‐speaking countries and may not relate closely to nursing care for people with DDs as practiced in other countries; however, we believe our recommendations can be relevant broadly to nursing educational and training settings.

## 5 CONCLUSION

Families with DDs face systemic disadvantages across the social determinants of health and interlocking barriers to health care that place them at a high risk for poor health outcomes. It is timely and crucial to generate evidence about effective strategies to educate, train and support nurses to develop their competencies in the delivery of quality health care for people with DDs in all sectors of the health and social care systems. We have highlighted some of the competencies (e.g., removing communication barriers) and professional supports (e.g., staff with skill mix) nurses need to care for people with DDs. We provide recommendations addressing access, education, collaboration, communication, use of standardized tools and creating a safe environment.

## AUTHOR CONTRIBUTIONS

Nazilla Khanlou conceptualized the project and design of the study and supervised all aspects of this work. Attia Khan implemented the research. Attia Khan and Luz Maria Vazquez contributed to the analysis of the results and to the writing of the manuscript. All authors discussed the results and contributed to the final manuscript.

## FUNDING INFORMATION

This work was supported by the Faculty of Health ‐ Minor Research Grant, York University, ON, Canada, granted to the first author (Nazilla Khanlou) as principal investigator. The fourth author (Rani Srivastava) was a coinvestigator on the project.

## CONFLICT OF INTEREST

No conflict of interest is declared by the authors.

## Data Availability

Data sharing is not applicable to this article as no new data were created or analyzed in this study.

## Endnotes

^1^
A law to include children with disabilities in public school settings and provide them free and appropriate services.

^2^
Social stories provide context of an experience in pictures / images to help a child with ASD understand what will happen for particular visits and to make the visit physically, socially and emotionally safe.
